# Sex Difference in the Association between Electronic Cigarette Use and Subsequent Cigarette Smoking among U.S. Adolescents: Findings from the PATH Study Waves 1–4

**DOI:** 10.3390/ijerph18041695

**Published:** 2021-02-10

**Authors:** Zongshuan Duan, Yu Wang, Jidong Huang

**Affiliations:** School of Public Health, Georgia State University, Atlanta, GA 30303, USA; zduan3@student.gsu.edu (Z.D.); ywang145@student.gsu.edu (Y.W.)

**Keywords:** adolescents, electronic cigarettes, cigarettes, sex difference, youth tobacco prevention

## Abstract

E-cigarettes are the most-used tobacco products among U.S. adolescents. Emerging evidence suggests that adolescents using e-cigarettes are at elevated risk for initiating cigarette smoking. However, whether this risk may differ by sex remains unknown. This study analyzed data from Wave 1 to 4 of the Population Assessment of Tobacco and Health (PATH) Study, a nationally representative longitudinal survey. Generalized estimation equations (GEE) were performed to estimate the associations between baseline e-cigarette use and subsequent cigarette smoking, controlling for sociodemographic characteristics, mental health conditions, and other tobacco use. Effect modifications by sex were examined. Multivariate analyses showed that, among baseline never cigarette smokers, past-30-day e-cigarette use at baseline waves was significantly associated with past-30-day cigarette smoking at follow-up waves (aOR = 3.90, 95% CI: 2.51–6.08). This association was significantly stronger for boys (aOR = 6.17, 95% CI: 2.43–15.68) than for girls (aOR = 1.10, 95% CI: 0.14–8.33). Additionally, using other tobacco products, older age, and having severe externalizing mental health problems at baseline were significantly associated with an increased likelihood of cigarette smoking at follow-up. The prospective association between e-cigarette use and cigarette smoking differs by sex among U.S. adolescents. Sex-specific tobacco control interventions may be warranted to curb the youth tobacco use epidemic.

## 1. Introduction

Cigarette smoking among U.S. youth has declined substantially since the mid-1990s [1]. Among 12th-grade students, the prevalence of past-30-day (P30D) cigarette smoking decreased steadily from 28.3% in 1996 to 7.5% in 2020 [2,3]. However, e-cigarettes are becoming increasingly popular among adolescents, including those who are not susceptible to smoking cigarettes [4,5,6]. In 2020, 19.6% of high school students and 4.7% of middle school students reported using e-cigarettes in the past 30 days [7]. A growing body of evidence documented a positive association between e-cigarette use and subsequent cigarette smoking initiation among tobacco-naïve adolescents [8,9,10], leading to concerns that the increased e-cigarette use among youth may potentially addict a new generation to combustible cigarettes, resulting in a lifetime nicotine addiction [4]. A recent meta-analysis combined the findings of 9 longitudinal studies and reported that, among youth and young adults, the pooled adjusted odds ratio (aOR) for subsequent cigarette smoking initiation was 3.62 (95% CI, 2.42–5.41) among ever e-cigarette users compared with never e-cigarette users. In addition, it found that the pooled aOR for P30D cigarette smoking at follow-up was 4.28 (95% CI, 2.52–7.27) among baseline P30D e-cigarette users compared with those who did not use e-cigarettes in the past 30 days at baseline [8].

Despite the growing number of studies investigating the prospective relationships between adolescent e-cigarette use and subsequent cigarette smoking, no study had examined how this association may differ by sex. The tobacco use behaviors and nicotine addiction among adolescents are dependent upon their sex [11]. Unfortunately, to date, tobacco control policies and interventions have remained largely sex blind, with limited recognition of the importance of understanding the sex differences in the mechanism and consequences of tobacco products’ initiation and transition [11,12]. Sex is a significant dimension for nearly all public health areas [13], and tobacco use is no exception. Evidence from laboratory experiments with non-human subjects indicated that the neurobiological mechanisms underlying nicotine seeking and metabolism differ between males and females [14,15]. Due to the presence of higher level of estrogen, females metabolize nicotine faster and experience lower rewarding effects of nicotine than males [16]. Therefore, while men were more likely to smoke for the reinforcing stimulant effects of nicotine, women were more likely to smoke for other reasons, such as emotion regulation and reaction to nicotine-related cues [17]. Additionally, a meta-analysis based on social studies revealed that the norms associated with adolescent boys and girls, and the sex composition of adolescents’ social networks might differentially affect their tobacco adoption and use behaviors [18]. In general, the traditional social norms placed more restrictions on women’s tobacco use behaviors, and the norms also contributed to social pressures and expectations against women’s smoking behaviors [18,19,20]. Due to variations in the factors contributing to sex differences in tobacco use, adolescent girls who use e-cigarettes may have different patterns of transitioning to cigarette smoking, compared with their male counterparts. Consequently, the general associations between e-cigarette use and subsequent cigarette initiation reported in previous studies may mask important sex differences, resulting in inaccurate predictions of the impacts of policies and interventions aiming to curb adolescent tobacco use.

In addition to the important knowledge gap regarding the potential sex difference, many previous studies did not control for the effect of mental health conditions, which were found to be associated with the initiation of tobacco use among adolescents [21,22,23]. Our study aims to address these critical research gaps. Specifically, we used the youth cohort from the first four waves of the Population Assessment of Tobacco and Health (PATH) Study to investigate whether the longitudinal association between initial e-cigarette use and subsequent cigarette smoking initiation would differ by sex, controlling for individual’s sociodemographic characteristics, use of other tobacco products, and mental health conditions. We hypothesize that this association would differ by sex. Additionally, we hypothesize that significant differences exist in cigarette smoking initiation between subgroups characterized by individual factors.

## 2. Materials and Methods

### 2.1. Study Sample and Design

Data were collected from 2013 through 2018 and analyzed in 2020. This study used a youth cohort sample (aged 12–17) compiled from Wave 1 (September 2013 to December 2014), Wave 2 (October 2014 to October 2015), Wave 3 (October 2015 to October 2016), and Wave 4 (December 2016 to January 2018) of the PATH Study, an ongoing nationally representative cohort study conducted by the U.S. National Institutes of Health (NIH) and the Food and Drug Administration (FDA) [24,25]. A four-stage stratified probability sample was selected to represent the noninstitutionalized population in the U.S. [25]. Among households that were screened, the weighted response rates for the youth cohort were 78.4% (Wave 1), 87.3% (Wave 2), 83.3% (Wave 3), and 79.5% (Wave 4), respectively [26,27]. In the PATH data, multiple imputations were performed on the variables such as sex, age, and use of tobacco products to address the missing data bias. Details regarding the PATH study design and sampling methods are published elsewhere [25], and are described in the PATH Study Public-Use Files user guide [26]. This study involved only secondary data analysis of the PATH survey data, which contained no personally identifiable information, and was exempt for ethical review by the Georgia State University Institutional Review Board (IRB Number: H20183; Reference Number: 357029). This article follows the reporting guideline for cohort studies of the Strengthening the Reporting of Observational Studies in Epidemiology (STROBE) [28].

In this study, we followed a validated approach used by the data management and research team of the PATH study to stack covariates in the baseline wave with cigarette smoking status at its corresponding follow-up wave study [29,30,31]. In this study, Wave 1, Wave 2, and Wave 3 were each considered as the baseline wave for its corresponding 12-month follow-up wave. The youth all-wave weights for the Wave 1 cohort were used to produce nationally representative estimates. The all-wave weights were assigned only to Wave 1 respondents and the shadow sample of individuals aged 9–11 at Wave 1 who completed interviews at all waves while they were 12–17 years old [27]. The target population of this study included youth respondents who reported never having used cigarettes at baseline waves. At each baseline wave, participants were asked, “Have you ever tried cigarette smoking, even one or two puffs”, and those who responded “No” were identified as baseline never cigarette smokers.

### 2.2. Measures

The primary outcome was the self-reported P30D cigarette smoking status at 12-month follow-up waves among never cigarette smokers at baseline waves. In the follow-up surveys, baseline never cigarette smokers who reported smoking at least one cigarette in the past 30 days at follow-up waves were coded as P30D cigarette smokers. Additionally, respondents who tried cigarette smoking, even one or two puffs in between baseline and 12-month follow-up, were defined as ever cigarette smokers.

The primary exposure variable was the P30D use of e-cigarettes at baseline waves. In each baseline wave, never cigarette smokers who reported having used any e-cigarettes in the past 30 days were categorized as baseline P30D users of e-cigarettes.

Covariates were potential confounding variables selected based on previous literature [8,10,29,32], including the following two domains. (1) Sociodemographic factors and other tobacco products use status at baseline waves: age (12–14 or 15–17), sex (male or female), race/ethnicity (Hispanic, Non-Hispanic White, Non-Hispanic Black, or Non-Hispanic Other), parental education (less than high school, high school graduate, some college or associate degree, and bachelor’s degree or above), and P30D use of other tobacco products (defined as using cigar, hookah, or smokeless tobacco in the past 30 days). Sexual orientation (straight vs. homosexual, bisexual, or other), which was only available for participants aged 14 and above, was used to examine the bivariate associations with outcome variables but not included in the regression analysis. (2) Intrapersonal factors: the internalizing and externalizing mental health problems over the past 12 months at baseline waves. The PATH study included four items measuring internalizing problems and seven items measuring externalizing problems (Table S1). In this study, we followed a validated approach to sum up the scores for internalizing and externalizing problems, where the severity of mental health problems was categorized into low (0–1), moderate (2–3), and high severity (4 for internalizing problems or 4–7 for externalizing problems) [33,34].

### 2.3. Statistical Analysis

All data management and analyses were conducted using Stata 15 (StataCorp LLC. College Station, TX, USA). The youth cohort all-wave weights were applied to account for the complex sample design features and to produce nationally representative estimates. The balanced repeated replication (BRR) approach with Fay’s adjustment of 0.3 was used to compute statistical precision for all estimations [25,29]. We reported the weighted prevalence of outcomes at follow-up waves and their weighted associations with covariates at baseline waves. Generalized estimating equations (GEE) with unstructured covariance were used to estimate the associations between the outcomes and exposure variables, controlling for individual sociodemographic characteristics, use of other tobacco products, and mental health conditions. Additional GEE models were fitted to examine the potential effect modifications of sex on the association between P30D e-cigarette use at baseline and P30D cigarette smoking at 12-month follow-up. Subgroup analyses, separately for adolescent boys and girls, were conducted to compare the associations between e-cigarette use and subsequent cigarette smoking. Additionally, sensitivity analyses, based on the same set of analyses mentioned above, in which the outcome measures were replaced with ever cigarette smoking, were conducted. All statistical tests were two-sided with the significance level set to 0.05.

## 3. Results

### 3.1. Sample and Demographic Characteristics

**Figure 1:**
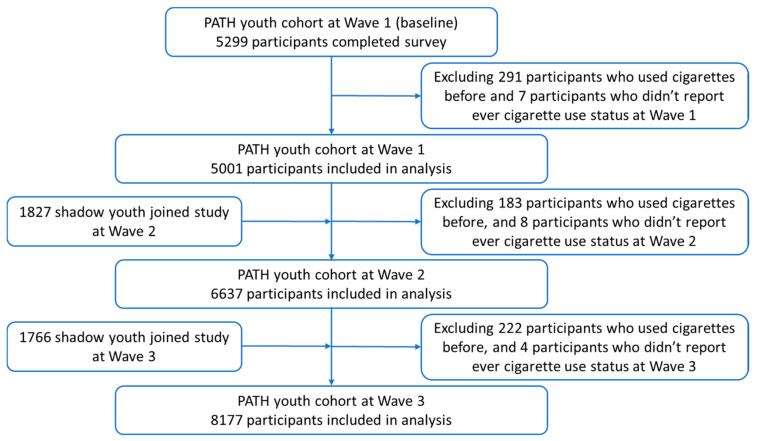
Flowchart for participants included in final analysis.

Our study sample included 5001 youth never cigarette smokers at Wave 1, 6637 at Wave 2, and 8177 at Wave 3. The enrollment and exclusion procedures are illustrated in Figure 1.

Among youth who reported never having used cigarettes at Wave 1, the weighted prevalence of P30D e-cigarette use was 0.4%; almost all (96.7%) respondents were between age 12 and 14; 49.2% of the respondents were girls; 53.4% were Non-Hispanic White, 14.4% were Non-Hispanic Black, and 23.0% were Hispanic; 0.4% used other tobacco products in the past 30 days; and 18.1% and 29.5% of them experienced high severity of internalizing and externalizing mental health problems in the past year, respectively. Among youth who reported never having used cigarettes at Wave 2, 0.9% of them used e-cigarette in the past 30 days; over three quarters (77.0%) of them were between age 12–14; 49.2% were girls; 52.5% were Non-Hispanic White, 13.9% were Non-Hispanic Black, and 23.9% were Hispanic; 0.6% used other tobacco products in the past 30 days; and 19.7% and 28.8% experienced high severity of internalizing and externalizing mental health problems in the past year, respectively. Among youth who reported never having used cigarettes at Wave 3, 1.5% used e-cigarette in the past 30 days; approximately two thirds of them were between age 12–14; 48.9% were girls; 51.7% were Non-Hispanic White, 13.6% were Non-Hispanic Black, and 24.6% were Hispanic; 0.7% used other tobacco products in the past 30 days; and 21.3% and 29.1% experienced high severity of internalizing and externalizing mental health problems in the past year, respectively. Detailed descriptive statistics of other characteristics are presented in Table 1.

### 3.2. Past-30-Day Cigarette Smoking at 12-Month Follow-up Waves

As shown in Table 2, among adolescents who reported P30D e-cigarette use at baseline waves, the prevalence of P30D cigarette smoking was 4.0% (95% CI: 0.5–27.7%) at Wave 2, 12.6% (95% CI: 5.1–27.6%) at Wave 3, and 9.1% (95% CI: 4.9–16.4%) at Wave 4, respectively. By contrast, among adolescents who did not use e-cigarette at baseline waves, the prevalence of P30D cigarette smoking was 1.2% (95% CI: 0.9–1.6%) at Wave 2, 0.8% (95% CI: 0.6–1.2%) at Wave 3, and 1.4% (95% CI: 1.1–1.7%) at Wave 4, respectively. In addition, generally, the weighted prevalence of self-reported P30D cigarette smoking at follow-up waves was higher among adolescents who were older, sexual minorities, and those having severe internalizing or externalizing mental health problems at baseline waves.

### 3.3. Multivariate Analyses

As shown in Table 3, after adjusting for individual characteristics, adolescents who reported P30D e-cigarette use at baseline waves were significantly more likely to report P30D cigarette smoking in the follow-up waves (aOR = 3.90, 95% CI: 2.51–6.08; *p* < 0.001) (Model 1). Older age, P30D use of other tobacco products, and severe externalizing mental health problems at the baseline waves were also statistically significantly associated with elevated odds of P30D cigarette smoking at follow-up waves, everything else being constant. In addition, being Non-Hispanic Black or Other and having parents with a bachelor’s degree or above were associated with reduced odds of P30D cigarette smoking at follow-up waves. As shown in Model 2, the interaction between P30D e-cigarette use and sex, noted as “P30D e-cigarette use # Sex,” was statistically significant (exponent of the estimated coefficients 3.18, 95% CI: 2.21–4.57), which indicated that the associations between cigarette smoking status at 12-month follow-up waves and P30D e-cigarette use at baseline waves were significantly different between adolescent boys and girls. 

Table 4 showed the results of the subgroup analyses stratified by sex. For boys, P30D cigarette smoking at 12-month follow-up waves was statistically significantly associated with P30D e-cigarette use at baseline waves (aOR = 6.17, 95% CI: 2.43–15.68; *p* < 0.001), controlling for individual characteristics. However, for girls, the corresponding association was not statistically significant (aOR = 1.10, 95% CI: 0.14–8.33; *p* = 0.154), controlling for other covariates. 

To evaluate whether the association between e-cigarette use and subsequent cigarette smoking is robust to outcome measures, sensitivity analyses in which cigarette smoking was measured by ever cigarette smoking, rather than P30D cigarette smoking at 12-month follow-up waves, were conducted. The results of the sensitivity analyses were presented in Tables S2–S4. Consistent with results presented in Table 2, results in Table S2 show that, at each follow-up wave, the prevalence of ever cigarette smoking was higher among adolescents who reported P30D e-cigarette use at corresponding baseline wave, compared with those who did not. In addition, results in Table S3 were similar to those in Table 3 regarding the adjusted associations between ever cigarette smoking at 12-month follow-up waves and characteristics at baseline waves. Notably, the interaction term between e-cigarette use and sex (P30D e-cigarette use # Sex) was also statistically significant, consistent with the results in Table 3. Furthermore, the results of subgroup analyses in Table S4 showed that the adjusted ORs between ever cigarette smoking at 12-month follow-up waves and P30D e-cigarette use at baseline waves were 5.81 (95% CI: 3.34–10.13; *p* < 0.001) and 2.31 (95% CI: 0.98–5.41; *p* = 0.052) for adolescent boys and girls, respectively.

## 4. Discussion

This study aimed to examine whether and to what extent sex would affect the associations between initial e-cigarette use and subsequent cigarette smoking among American adolescents (aged 12–17). Although the longitudinal associations between e-cigarette use and subsequent cigarette smoking had been documented [8,9,10], no previous studies examined the potential difference in this relationship by sex. Our results revealed, consistent with previous studies, that e-cigarettes use at baseline waves was significantly associated with P30D cigarette smoking at 12-month follow-up waves. More importantly, our study added to the current knowledge base by revealing that this association was significantly stronger for boys than for girls. The differential patterns were consistently observed regardless of whether the follow-up cigarette smoking status was measured by past 30-day use or ever use. The consistency indicates that sex differences in the association between initial P30D e-cigarette use and subsequent cigarette smoking are robust to outcome measures.

The differential effects characterized by sex may be partially attributable to the different levels of nicotine dependence between boys and girls. Our study showed that among P30D e-cigarette users, the number of days using e-cigarettes in the past 30 days were higher for boys than for girls (Table S5). The difference may indicate that among adolescents who used e-cigarettes, the level of nicotine dependence was likely to be higher for boys than girls. This finding is consistent with a recent literature review concluding that boys tended to use e-cigarettes more frequently than girls [35]. Since youth with higher nicotine dependence levels were presumably more likely to transition to cigarette smoking, the difference in nicotine dependence between e-cigarette using boys and girls may explain why e-cigarette using boys were more likely to advance to cigarette smoking than e-cigarette using girls [10]. In addition to use frequency, several other potential reasons may explain why boys may be more likely to develop nicotine dependency than girls from vaping. First, evidence showed that females metabolized nicotine faster than males due to estrogen [16]. The differential metabolism rates by sex suggests that females are more likely to experience higher adverse sensitivity and lower rewarding effects of nicotine than their male counterparts [36,37,38]. Consequently, e-cigarette using girls may be less likely to develop nicotine dependency and less susceptible to transition to cigarette smoking. In addition, the sources of acquisition for e-cigarettes may be different between girls and boys. A study in Connecticut showed that compared with boys, girls were more likely to obtain e-cigarettes from their peers [39], suggesting more social and less frequent e-cigarette use among girls; hence, the difference in transitioning to cigarette smoking. The sex differences in the association between baseline e-cigarette use and subsequent cigarette initiation suggest that policies/interventions aiming to combat the youth vaping epidemic may reduce subsequent cigarette smoking among the U.S. youth population, particularly among adolescent boys. For example, a vaping cessation media campaign that specifically targets at boys may reduce e-cigarette use among boys, and consequently, making them less likely to transition from e-cigarettes to cigarettes.

Our results also show that internalizing and externalizing mental health problems were prospectively associated with cigarette smoking initiation, controlling for sociodemographic covariates and use of e-cigarettes and other tobacco products. Our findings were consistent with other published studies [21,40], suggesting that a wide range of mental health problems could be considered as predictors of cigarette smoking among adolescents. Early screening for mental health problems combined with targeted mental health interventions (e.g., school counseling, preventive efforts through primary care providers) may help reduce cigarette smoking among vulnerable youth [41,42].

Additionally, we found that older age, being Non-Hispanic White, and using other tobacco products were significantly associated with subsequent cigarette smoking in our study, consistent with findings reported in previous studies [8,10,29,32]. Notably, the magnitude of the association between P30D e-cigarette use at baseline waves and subsequent cigarette smoking was comparable to the association between P30D other tobacco use at baseline waves and subsequent cigarette smoking, indicating the importance of e-cigarette use in predicting subsequent cigarette smoking among U.S. adolescents. Continued surveillance of e-cigarette and other tobacco product use among youth is, therefore, warranted. 

This study is subject to several limitations. First, self-reported use of e-cigarettes, cigarettes, and other tobacco products may introduce recall bias and social desirability bias [43]. Second, the small sample size of adolescent e-cigarette users in the PATH study prevented us from conducting a mediation analysis to further examine whether the sex differences could be partially attributed to different nicotine dependence levels between boys and girls. Future studies are needed to explore the mechanisms of the differential effects characterized by sex and other potential characteristics. Third, the association between e-cigarette use at baseline waves and subsequent cigarette smoking identified in this study did not represent a true causal relationship. However, our study did control for a wide range of potential confounding factors and established a temporal relationship and chronological sequence between e-cigarette use and subsequent cigarette smoking, addressing most, if not all, of the concerns and criticisms on the current literature regarding the potential gateway effect of e-cigarettes to cigarette smoking [5,44,45,46]. 

## 5. Conclusions

This study’s findings highlighted the important sex differences in the longitudinal association between initial e-cigarette use and subsequent cigarette smoking among U.S. adolescents. Efforts to curb the adolescent vaping epidemic may have added benefits to reduce cigarette initiation, particularly among adolescent boys. Sex-specific tobacco control interventions may be warranted to reduce youth tobacco use. In addition, targeted tobacco control interventions, focusing on youth with severe mental health conditions, are warranted. Finally, continued efforts are needed to monitor tobacco and cigarette transitions among youth, particularly among vulnerable and high-risk youth subpopulations.

## Figures and Tables

**Table 1:** Descriptive statistics of covariates at baseline waves among adolescents (12–17 years of age) who reported never having smoked cigarettes.

	Wave 1 (*n* = 5001)	Wave 2 (*n* = 6637)	Wave 3 (*n* = 8177)
	*n* (%)	*n* (%)	*n* (%)
Interview status			
Youth (aged 12–17)	5001 (100)	4864 (73.3)	4711 (57.6)
Shadow youth (aged 9–11)	0 (0)	1773 (26.7)	3466 (42.4)
P30D e-cigarette use			
Yes	19 (0.4)	53 (0.9)	112 (1.5)
No	4949 (99.6)	6538 (99.1)	8033 (98.5)
Age group			
12–14	4388 (96.7)	5147 (77.0)	5175 (63.3)
15–17	168 (3.3)	1490 (23.0)	3002 (36.7)
Sex			
Male	2551 (50.8)	3365 (50.8)	4190 (51.1)
Female	2450 (49.2)	3253 (49.2)	3963 (48.9)
Race/ethnicity			
Non-Hispanic White	2334 (53.4)	2984 (52.5)	3624 (51.7)
Non-Hispanic Black	722 (14.4)	899 (13.9)	1087 (13.6)
Non-Hispanic Other	447 (9.2)	586 (9.7)	739 (10.1)
Hispanic	1498 (23.0)	1946 (23.9)	2402 (24.6)
Sexual orientation (ages 14+)			
Straight/Heterosexual	1455 (94.9)	2867 (92.3)	4145 (90.9)
Gay, lesbian, bisexual, or other	75 (5.1)	234 (7.7)	421 (9.1)
Parental education			
Less than high school	1009 (17.4)	1199 (16.4)	1511 (15.9)
High school graduate	907 (17.3)	1105 (17.0)	1392 (16.4)
Some college or associate degree	1024 (19.9)	1882 (30.3)	2519 (31.0)
Bachelor’s degree or above	2032 (45.4)	1928 (36.6)	2583 (36.7)
P30D use of other tobacco products ^1^			
Yes	19 (0.4)	32 (0.6)	56 (0.7)
No	4757 (99.6)	6461 (99.4)	8059 (99.3)
Past year internalizing problems			
Low	2558 (52.5)	3420 (52.6)	4023 (50.7)
Moderate	1432 (29.4)	1771 (27.7)	2205 (28.0)
High	856 (18.1)	1269 (19.7)	1704 (21.3)
Past year externalizing problems			
Low	1901 (39.8)	2776 (43.4)	3397 (43.2)
Moderate	1446 (30.7)	1738 (27.8)	2127 (27.8)
High	1368 (29.5)	1807 (28.8)	2249 (29.1)

^1^ Other tobacco included cigars (traditional cigars, cigarillos, or filtered cigars), hookah, and smokeless tobacco (snus pouches, loose snus, moist snuff, dip, spit, or chewing tobacco).

**Table 2:** Percentage of past-30-day (P30D) cigarette smoking at each follow-up wave by covariates at its corresponding baseline wave among baseline never cigarette smokers.

Covariates at Corresponding Baseline Wave	P30D Cigarette Smoking at Follow-up Waves		
	Wave 2 (*n* = 5001)	Wave 3 (*n* = 6637)	Wave 4 (*n* = 8177)
	% (95% CI)	% (95% CI)	% (95% CI)
Total	1.2 (0.9–1.6)	0.9 (0.7–1.2)	1.5 (1.2–1.8)
P30D e-cigarette use			
Yes	4.0 (0.5–27.7)	12.6 (5.1–27.6)	9.1 (4.9–16.4)
No	1.2 (0.9–1.6)	0.8 (0.6–1.2)	1.4 (1.1–1.7)
Age group			
12–14	1.1 (0.8–1.6)	0.7 (0.5–1.1)	0.9 (0.6–1.2)
15–17	1.9 (0.6–5.9)	1.7 (1.2–2.5)	2.6 (2.0–3.5)
Sex			
Male	0.8 (0.5–1.3)	0.6 (0.4–1.0)	1.7 (1.3–2.1)
Female	1.6 (1.1–2.2)	1.3 (0.9–1.8)	1.4 (1.0–1.9)
Race/ethnicity			
Non-Hispanic White	1.3 (0.9–2.0)	1.3 (0.9–1.8)	1.9 (1.4–2.5)
Non-Hispanic Black	0.5 (0.2–1.7)	0.3 (0.1–0.8)	0.8 (0.4–1.7)
Non-Hispanic Other	1.1 (0.4–3.2)	1.1 (0.4–2.9)	1.0 (0.5–1.8)
Hispanic	1.2 (0.7–2.0)	0.6 (0.3–1.1)	1.4 (1.0–2.0)
Sexual orientation (ages 14+)			
Straight/Heterosexual	1.7 (1.0–2.7)	1.0 (0.7–1.6)	2.0 (1.5–2.5)
Gay, lesbian, bisexual, or other	4.7 (1.6–12.9)	3.3 (1.5–7.1)	5.3 (3.4–8.3)
Parental education			
Less than high school	1.5 (0.8–2.8)	0.6 (0.3–1.5)	2.1 (1.4–3.0)
High school graduate	1.7 (0.9–3.0)	1.4 (1.7–2.8)	1.4 (0.9–2.2)
Some college or associate degree	1.9 (1.1–3.0)	0.8 (0.5–1.3)	1.8 (1.3–2.5)
Bachelor’s degree or above	0.6 (0.3–1.0)	0.8 (0.5–1.4)	1.1 (0.7–1.7)
P30D use of other tobacco products ^1^			
Yes	4.5 (0.5–30.8)	8.7 (2.2–29.0)	12.7 (6.1–24.8)
No	1.2 (0.9–1.6)	0.9 (0.7–1.2)	1.4 (1.2–1.8)
Past year internalizing problems			
Low	0.7 (0.4–1.2)	0.6 (0.4–1.0)	1.3 (0.9–1.8)
Moderate	1.5 (0.9–2.5)	1.2 (0.7–2.0)	1.1 (0.7–1.7)
High	1.8 (1.1–3.0)	1.4 (0.8–2.4)	2.6 (1.9–3.5)
Past year externalizing problems			
Low	0.4 (0.2–0.9)	0.6 (0.4–1.0)	1.1 (0.8–1.6)
Moderate	1.1 (0.6–2.0)	0.8 (0.4–1.6)	1.2 (0.8–1.9)
High	2.5 (1.7–3.6)	1.5 (0.9–2.4)	2.2 (1.6–2.9)

^1^ Other tobacco included cigars (traditional cigars, cigarillos, or filtered cigars), hookah, and smokeless tobacco (snus pouches, loose snus, moist snuff, dip, spit, or chewing tobacco).

**Table 3:** Adjusted odds ratios (aORs) of P30D cigarette smoking at 12-month follow-up waves among adolescents (12–17 years of age) who were never cigarette smokers at baseline waves.

	Model 1	Model 2
	No Interaction	With Interaction
	aOR (95% CI)	aOR (95% CI)
P30D e-cigarette use		
Yes	3.90 (2.51–6.08)	1.93 (0.79–4.71)
No	Ref.	Ref.
Sex		
Male	1.24 (1.03–1.49)	1.19 (0.98–1.43)
Female	Ref.	Ref.
P30D e-cigarette use # Sex		
Yes # Male		3.18 (2.21–4.57)
No # Female		Ref.
Age group		
12–14	Ref.	Ref.
15–17	1.80 (1.44–2.26)	1.81 (1.44–2.26)
Race/ethnicity		
Non-Hispanic White	Ref.	Ref.
Non-Hispanic Black	0.46 (0.30–0.70)	0.46 (0.30–0.70)
Non-Hispanic Other	0.66 (0.43–1.01)	0.66 (0.43–1.01)
Hispanic	0.66 (0.50–0.89)	0.66 (0.50–0.89)
Parental education		
Less than high school	Ref.	Ref.
High school graduate	0.92 (0.66–1.29)	0.92 (0.65–1.29)
Some college or associate degree	0.76 (0.55–1.05)	0.75 (0.54–1.04)
Bachelor’s degree or above	0.50 (0.35–0.71)	0.50 (0.35–0.71)
P30D use of other tobacco products ^1^		
Yes	3.22 (1.23–8.46)	3.45 (1.36–8.70)
No	Ref.	Ref.
Internalizing mental health problems		
Low	Ref.	Ref.
Moderate	1.33 (1.04–2.58)	1.33 (1.05–1.69)
High	1.90 (1.40–2.58)	1.93 (1.42–2.63)
Externalizing mental health problems		
Low	Ref.	Ref.
Moderate	1.40 (1.01–1.95)	1.41 (1.01–1.97)
High	2.11 (1.55–2.88)	2.09 (1.54–2.85)

^1^ Other tobacco included cigars (traditional cigars, cigarillos, or filtered cigars), hookah, and smokeless tobacco (snus pouches, loose snus, moist snuff, dip, spit, or chewing tobacco). Ref.: reference group.

**Table 4:** Adjusted odds ratios (aORs) ^1^ from subgroup analysis for adolescent boys and girls.

	Boys P30D Cigarette Smoking	Girls P30D Cigarette Smoking
	aOR (95% CI)	aOR (95% CI)
P30D e-cigarette use		
Yes	6.17 (2.43–15.68)	1.10 (0.14–8.33)
No	Ref.	Ref.

^1^ Controlling for age, race/ethnicity, parental education, P30D other tobacco use, past-year internalizing mental health problems, and past-year externalizing mental health problems. Ref.: reference group.

## Data Availability

Publicly available datasets were analyzed in this study. This data can be found here: [https://www.icpsr.umich.edu/icpsrweb/NAHDAP/search/studies?q=PATH (accessed on 20 March 2020)].

## Supplementary Materials

The following are available online at https://www.mdpi.com/1660-4601/18/4/1695/s1, Table S1: Items for internalizing and externalizing mental health problems, Table S2: Percentage of ever cigarette smoking at each follow-up wave by covariates at its corresponding baseline wave among baseline never cigarette smokers, Table S3: Adjusted odds ratios (aORs) of ever cigarette smoking at 12-month follow-up waves among adolescents (12–17 years of age) who were never cigarette smokers at baseline waves, Table S4: Adjusted odds ratios (aORs) from subgroup analysis for adolescent boys and girls, Table S5: E-cigarette dependence among P30D e-cigarette users for adolescent boys and girls (aged 12–17).

## References

[1] U.S. Department of Health and Human Services (2014). The Health Consequences of Smoking—50 Years of Progress: A Report of the Surgeon General.

[2] Johnston L.D., Miech R.A., O’Malley P.M., Bachman J.G., Schulenberg J.E., Patrick M.E. (2019). Monitoring the Future National Survey Results on Drug Use, 1975–2018: Overview, Key Findings on Adolescent Drug Use.

[3] National Institute on Drug Abuse Study: Surge of Teen Vaping Levels off, but Remains High as of Early 2020. https://www.drugabuse.gov/news-events/news-releases/2020/12/study-surge-of-teen-vaping-levels-off-but-remains-high-as-of-early-2020.

[4] U.S. Department of Health and Human Services (2016). E-Cigarette Use among Youth and Young Adults: A Report of the Surgeon General.

[5] Khouja J.N., Suddell S.F., Peters S.E., Taylor A.E., Munafò M.R. (2020). Is e-cigarette use in non-smoking young adults associated with later smoking? A systematic review and meta-analysis. Tob. Control.

[6] Huang J., Duan Z., Kwok J., Binns S., Vera L.E., Kim Y., Szczypka G., Emery S.L. (2019). Vaping versus JUULing: How the extraordinary growth and marketing of JUUL transformed the US retail e-cigarette market. Tob. Control.

[7] Wang T.W., Neff L.J., Park-Lee E., Ren C., Cullen K.A., King B.A. (2020). E-cigarette use among middle and high school students—United States, 2020. Morb. Mortal. Wkly. Rep..

[8] Soneji S., Barrington-Trimis J.L., Wills T.A., Leventhal A.M., Unger J.B., Gibson L.A., Yang J., Primack B.A., Andrews J.A., Miech R.A. (2017). Association between initial use of e-cigarettes and subsequent cigarette smoking among adolescents and young adults: A systematic review and meta-analysis. JAMA Pediatr..

[9] Leventhal A.M., Strong D.R., Kirkpatrick M.G., Unger J.B., Sussman S., Riggs N.R., Stone M.D., Khoddam R., Samet J.M., Audrain-McGovern J. (2015). Association of electronic cigarette use with initiation of combustible tobacco product smoking in early adolescence. JAMA.

[10] Primack B.A., Soneji S., Stoolmiller M., Fine M.J., Sargent J.D. (2015). Progression to traditional cigarette smoking after electronic cigarette use among US adolescents and young adults. JAMA Pediatr..

[11] Amos A., Greaves L., Nichter M., Bloch M. (2012). Women and tobacco: A call for including gender in tobacco control research, policy and practice. Tob. Control.

[12] Solomon A. (2020). Gender, women, and the future of tobacco control. Drugs Alcohol Today.

[13] Regitz-Zagrosek V. (2012). Sex and gender differences in health: Science & Society Series on Sex and Science. EMBO Rep..

[14] Pogun S., Yararbas G. (2009). Sex differences in nicotine action. Nicotine Psychopharmacol..

[15] Schmidt H.D., Rupprecht L.E., Addy N.A. (2018). Neurobiological and neurophysiological mechanisms underlying nicotine seeking and smoking relapse. Mol. Neuropsychiatry.

[16] Benowitz N.L., Lessov-Schlaggar C.N., Swan G.E., Jacob P. (2006). Female sex and oral contraceptive use accelerate nicotine metabolism. Clin. Pharmacol. Ther..

[17] Cosgrove K.P., Wang S., Kim S.-J., McGovern E., Nabulsi N., Gao H., Labaree D., Tagare H.D., Sullivan J.M., Morris E.D. (2014). Sex differences in the brain’s dopamine signature of cigarette smoking. J. Neurosci..

[18] Jacobs W., Goodson P., Barry A.E., McLeroy K.R. (2016). The role of gender in adolescents’ social networks and alcohol, tobacco, and drug use: A systematic review. J. Sch. Health.

[19] Waldron I. (1991). Patterns and causes of gender differences in smoking. Soc. Sci. Med..

[20] Piper M.E., Cook J.W., Schlam T.R., Jorenby D.E., Smith S.S., Bolt D.M., Loh W.-Y. (2010). Gender, race, and education differences in abstinence rates among participants in two randomized smoking cessation trials. Nicotine Tob. Res..

[21] Riehm K.E., Young A.S., Feder K.A., Krawczyk N., Tormohlen K.N., Pacek L.R., Mojtabai R., Crum R.M. (2019). Mental health problems and initiation of e-cigarette and combustible cigarette use. Pediatrics.

[22] Upadhyaya H.P., Deas D., Brady K.T., Kruesi M. (2002). Cigarette smoking and psychiatric comorbidity in children and adolescents. J. Am. Acad. Child Adolesc. Psychiatry.

[23] Audrain-McGovern J., Rodriguez D., Kassel J.D. (2009). Adolescent smoking and depression: Evidence for self-medication and peer smoking mediation. Addiction.

[24] National Institute on Drug Abuse, Food and Drug Administration Center for Tobacco Products (2020). Population Assessment of Tobacco and Health (PATH) Study [United States] Public-Use Files. Inter-University Consortium for Political and Social Research [Distributor].

[25] Hyland A., Ambrose B.K., Conway K.P., Borek N., Lambert E., Carusi C., Taylor K., Crosse S., Fong G.T., Cummings K.M. (2017). Design and methods of the Population Assessment of Tobacco and Health (PATH) Study. Tob. Control.

[26] Inter-University Consortium for Political and Social Research Population Assessment of Tobacco and Health (PATH) Study [United States] Public-Use Files ICPSR Public-Use Files User Guide. https://www.icpsr.umich.edu/files/NAHDAP/documentation/ug36498-all.pdf.

[27] National Institute on Drug Abuse, Food Drug Administration Center for Tobacco Products (2020). Population Assessment of Tobacco and Health (PATH) Study [United States] Restricted-Use Files. Inter-university Consortium for Political and Social Research [distributor].

[28] Von Elm E., Altman D.G., Egger M., Pocock S.J., Gøtzsche P.C., Vandenbroucke J.P. (2007). The Strengthening the Reporting of Observational Studies in Epidemiology (STROBE) statement: Guidelines for reporting observational studies. Ann. Intern. Med..

[29] Kasza K.A., Edwards K.C., Tang Z., Stanton C.A., Sharma E., Halenar M.J., Taylor K.A., Donaldson E., Hull L.C., Day H. (2020). Correlates of tobacco product initiation among youth and adults in the USA: Findings from the PATH Study Waves 1–3 (2013–2016). Tob. Control.

[30] Kasza K.A., Edwards K.C., Tang Z., Stanton C.A., Sharma E., Halenar M.J., Taylor K.A., Donaldson E.A., Hull L.C., Bansal-Travers M. (2020). Correlates of tobacco product cessation among youth and adults in the USA: Findings from the PATH Study Waves 1–3 (2013–2016). Tob. Control.

[31] Edwards K.C., Kasza K.A., Tang Z., Stanton C.A., Sharma E., Halenar M.J., Taylor K.A., Donaldson E.A., Hull L.C., Bansal-Travers M. (2020). Correlates of tobacco product reuptake and relapse among youth and adults in the USA: Findings from the PATH Study Waves 1–3 (2013–2016). Tob. Control.

[32] Stanton C.A., Sharma E., Seaman E.L., Kasza K.A., Edwards K.C., Halenar M.J., Taylor K.A., Day H., Anic G., Hull L.C. (2020). Initiation of any tobacco and five tobacco products across 3 years among youth, young adults and adults in the USA: Findings from the PATH Study Waves 1–3 (2013–2016). Tob. Control.

[33] Conway K.P., Green V.R., Kasza K.A., Silveira M.L., Borek N., Kimmel H.L., Sargent J.D., Stanton C., Lambert E., Hilmi N. (2017). Co-occurrence of tobacco product use, substance use, and mental health problems among adults: Findings from Wave 1 (2013–2014) of the Population Assessment of Tobacco and Health (PATH) Study. Drug Alcohol Depend..

[34] Conway K.P., Green V.R., Kasza K.A., Silveira M.L., Borek N., Kimmel H.L., Sargent J.D., Stanton C.A., Lambert E., Hilmi N. (2018). Co-occurrence of tobacco product use, substance use, and mental health problems among youth: Findings from wave 1 (2013–2014) of the population assessment of tobacco and health (PATH) study. Addict. Behav..

[35] Kong G., Kuguru K.E., Krishnan-Sarin S. (2017). Gender differences in US adolescent e-cigarette use. Curr. Addict. Rep..

[36] Perkins K.A. (2009). Acute responses to nicotine and smoking: Implications for prevention and treatment of smoking in lower SES women. Drug Alcohol Depend..

[37] Sofuoglu M., Mooney M. (2009). Subjective responses to intravenous nicotine: Greater sensitivity in women than in men. Exp. Clin. Psychopharmacol..

[38] Rubinstein M.L., Shiffman S., Rait M.A., Benowitz N.L. (2013). Race, gender, and nicotine metabolism in adolescent smokers. Nicotine Tob. Res..

[39] Kong G., Morean M.E., Cavallo D.A., Camenga D.R., Krishnan-Sarin S. (2017). Sources of electronic cigarette acquisition among adolescents in Connecticut. Tob. Regul. Sci..

[40] Green V.R., Conway K.P., Silveira M.L., Kasza K.A., Cohn A., Cummings K.M., Stanton C.A., Callahan-Lyon P., Slavit W., Sargent J.D. (2018). Mental health problems and onset of tobacco use among 12-to 24-year-olds in the PATH study. J. Am. Acad. Child Adolesc. Psychiatry.

[41] Flay B.R. (2009). School-based smoking prevention programs with the promise of long-term effects. Tob. Induc. Dis..

[42] Kulig J.W. (2005). Tobacco, alcohol, and other drugs: The role of the pediatrician in prevention, identification, and management of substance abuse. Pediatrics.

[43] Coughlin S.S. (1990). Recall bias in epidemiologic studies. J. Clin. Epidemiol..

[44] Chapman S., Bareham D., Maziak W. (2019). The gateway effect of e-cigarettes: Reflections on main criticisms. Nicotine Tob. Res..

[45] Shahab L., Beard E., Brown J. (2020). Association of initial e-cigarette and other tobacco product use with subsequent cigarette smoking in adolescents: A cross-sectional, matched control study. Tob. Control.

[46] Morgenstern M., Nies A., Goecke M., Hanewinkel R. (2018). E-Cigarettes and the Use of Conventional Cigarettes: A cohort study in 10th grade students in Germany. Dtsch. Ärzteblatt Int..

