# Supplementary material for: Sex Difference in the Association between Electronic Cigarette Use and Subsequent Cigarette Smoking among U.S. Adolescents: Findings from the PATH Study Waves 1–4
Source: Int J Environ Res Public Health. 2021 Feb 10;18(4):1695. doi: 10.3390/ijerph18041695 (PMC7916485; doi:10.3390/ijerph18041695)
Supplement: Supplementary file 1 [file ijerph-18-01695-s001.pdf]

International Journal of
Environmental Research and Public Health

MDPI

Table S1. Items for internalizing and externalizing mental health problems.

GAIN-SS Subscale [1]	Items
Internalizing mental health problems	Feeling very trapped, lonely, sad, blue, depressed, or hopeless about the future
	Sleep trouble - such as bad dreams, sleeping restlessly or falling asleep during the day
	Feeling very anxious, nervous, tense, scared, panicked or like something bad was going to happen
	Becoming very distressed and upset when something reminded you of the past
Externalizing mental health problems	Lied or conned to get things you wanted or to avoid having to do something
	Had a hard time paying attention at school, work, or home
	Had a hard time listening to instructions at school, work, or home
	Were a bully or threatened other people
	Started physical fights with other people
	Felt restless or the need to run around or climb on things
	Gave answers before the other person finished asking the question

[1] GAIN-SS: Global Appraisal of Individual Needs – Short Screener.

Table S2. Percentage of ever cigarette smoking at each follow-up wave by covariates at its corresponding baseline wave among baseline never cigarette smokers.

	Wave 2 (*n* = 5001)	Wave 3 (*n* = 6637)	Wave 4 (*n* = 8177)
	% (95% CI)	% (95% CI)	% (95% CI)
P30D e-cigarette use			
Yes	12.8 (3.6 – 36.5)	25.6 (14.2 – 41.9)	18.9 (12.4 – 27.7)
No	2.8 (2.3 – 3.3)	2.7 (2.3 – 3.1)	3.2 (2.7 – 3.6)
Age group			
12–14	2.8 (2.3 – 3.4)	2.3 (2.0 – 2.8)	2.3 (1.9 – 2.8)
15–17	2.9 (1.2 – 7.0)	4.6 (3.6 – 5.9)	5.3 (4.4 – 6.3)
Sex			
Male	2.6 (1.9 – 3.5)	2.8 (2.2 – 3.5)	3.8 (3.2 – 4.4)
Female	3.1 (2.5 – 3.8)	3.0 (2.4 – 3.6)	3.0 (2.5 – 3.7)
Race/ethnicity			
Non-Hispanic White	3.1 (2.4 – 3.9)	3.7 (3.0 – 4.5)	3.9 (3.2 – 4.8)
Non-Hispanic Black	2.6 (1.5 – 4.4)	1.4 (0.8 – 2.6)	2.1 (1.3 – 3.3)
Non-Hispanic Other	1.6 (0.7 – 3.6)	2.7 (1.5 – 4.7)	2.5 (1.7 – 3.8)
Hispanic	2.8 (2.0 – 3.8)	2.3 (1.6 – 3.2)	3.3 (2.6 – 4.4)
Sexual orientation (ages 14+)			
Straight/Heterosexual	3.6 (2.7 – 4.8)	3.5 (2.8 – 4.4)	4.2 (3.6 – 5.0)
Gay, lesbian, bisexual, or other	10.8 (4.5 – 23.8)	9.4 (5.8 – 14.8)	9.8 (7.3 – 13.2)
Parental education			
Less than high school	4.1 (2.9 – 5.8)	2.6 (1.8 – 3.8)	4.1 (3.0 – 5.5)
High school graduate	3.3 (2.2 – 4.9)	4.2 (3.0 – 5.9)	3.7 (2.7 – 5.1)
Some college or associate degree	2.9 (2.0 – 4.1)	3.2 (2.5 – 4.1)	3.7 (3.1 – 4.6)
Bachelor's degree or above	2.1 (1.5 – 2.9)	2.1 (1.5 – 2.9)	2.7 (2.1 – 3.4)
P30D use of other tobacco products [1]			
Yes	4.5 (0.5 – 30.8)	8.7 (2.2 – 29.0)	21.9 (11.8 – 37.0)
No	2.8 (2.3 – 3.4)	2.8 (2.4 – 3.3)	3.2 (2.8 – 3.7)
Past year internalizing problems			
Low	1.4 (0.9 – 2.1)	1.8 (1.3 – 2.5)	2.6 (2.11 – 3.2)
Moderate	3.7 (2.8 – 5.0)	2.9 (2.1 – 4.0)	2.8 (2.0 – 4.0)

High	5.2 (4.0 − 6.8)	5.7 (4.3 − 7.4)	5.8 (4.8 − 7.1)
Past year externalizing problems			
Low	0.6 (0.3 − 1.2)	1.9 (1.4 − 2.6)	2.4 (1.8 − 3.1)
Moderate	3.1 (2.3 − 4.3)	2.5 (1.8 − 3.5)	3.2 (2.5 − 4.2)
High	5.8 (4.6 − 7.3)	4.6 (3.5 − 5.9)	5.0 (4.0 − 6.1)

[1] Other tobacco included cigars (traditional cigars, cigarillos, or filtered cigars), hookah, and smokeless tobacco (snus pouches, looseijerph18041695 ijerph18041695 ijerph18041695 snus, moist snuff, dip, spit, or chewing tobacco).

Table S3. Adjusted odds ratios (aORs) of ever cigarette smoking at 12-month follow-up waves among adolescents (12–17 years of age) who were never cigarette smokers at baseline waves.

	**Model 1**	**Model 2**
	**No Interaction aOR (95% CI)**	**With Interaction aOR (95% CI)**
P30D e-cigarette use		
Yes	3.17 (1.45 − 6.94)	0.86 (0.12 − 6.43)
No	Ref.	Ref.
Sex		
Male	0.84 (0.62 − 1.14)	0.77 (0.56 − 1.04)
Female	Ref.	Ref.
P30D e-cigarette use # Sex		
Yes # Male		7.78 (5.01 − 12.08)
No # Female		Ref.
Age group		
12–14	Ref.	Ref.
15–17	2.34 (1.63 − 3.36)	2.34 (1.63 − 3.35)
Race/ethnicity		
Non-Hispanic White	Ref.	Ref.
Non-Hispanic Black	0.31 (0.15 − 0.64)	0.30 (0.14 − 0.63)
Non-Hispanic Other	0.71 (0.38 − 1.32)	0.71 (0.38 − 1.32)
Hispanic	0.53 (0.34 − 0.84)	0.53 (0.33 − 0.83)
Parental education		
Less than high school	Ref.	Ref.
High school graduate	0.79 (0.47 − 1.34)	0.78 (0.47 − 1.32)
Some college or associate degree	0.79 (0.48 − 1.30)	0.77 (0.47 − 1.27)
Bachelor's degree or above	0.41 (0.24 − 0.69)	0.40 (0.24 − 0.67)
P30D use of other tobacco products [1]		
Yes	5.07 (1.48 − 17.42)	5.73 (1.75 − 18.73)
No	Ref.	Ref.
Internalizing mental health problems		
Low	Ref.	Ref.
Moderate	1.07 (0.65 − 1.75)	1.08 (0.66 − 1.77)
High	1.19 (0.73 − 1.94)	1.23 (0.75 − 2.02)
Externalizing mental health problems		
Low	Ref.	Ref.
Moderate	1.33 (0.84 − 2.11)	1.35 (0.85 − 2.16)
High	2.56 (1.64 − 4.00)	2.53 (1.63 − 3.92)

[1] Other tobacco included cigars (traditional cigars, cigarillos, or filtered cigars), hookah, and smokeless tobacco (snus pouches, loose snus, moist snuff, dip, spit, or chewing tobacco). Ref.: reference group.

Table S4. Adjusted ORs [1] from subgroup analysis for adolescent boys and girls.

	Boys ever Cigarette Smoking	Girls ever Cigarette Smoking
	aOR (95% CI)	aOR (95% CI)
P30D e-cigarette use		
Yes	5.81 (3.34 – 10.13)	2.31 (0.98 – 5.41)
No	Ref.	Ref.

[1] Controlling for age, race/ethnicity, parental education, P30D other tobacco use, past-year internalizing mental health problems, and past-year externalizing mental health problems. Ref.: reference group.

Table S5. E-cigarette dependence among P30D e-cigarette users for adolescent boys and girls (aged 12–17).

	Wave 1 (*n* = 29)	Wave 2 (*n* = 54)	Wave 3 (*n* = 97)
	n (mean/%)	n (mean/%)	n (mean/%)
Number of days using an e-cigarette in P30D (mean)			
Overall	29 (4.1)	54 (4.3)	97 (7.5)
Male	15 (5.0)	30 (5.6)	60 (8.4)
Female	14 (3.1)	24 (2.9)	37 (6.1)
E-cigarette dependence [1] (%)			
Low	27 (93.4)	52 (97.3)	82 (83.1)
High	2 (6.6)	2 (2.7)	15 (16.9)
E-cigarette dependence for boys (%)			
Low	13 (87)	28 (94.8)	49 (81.9)
High	2 (13)	2 (5.2)	11 (18.1)
E-cigarette dependence for girls (%)			
Low	14 (100)	24 (100)	33 (85.2)
High	0 (0)	0 (0)	4 (14.8)

[1] Nicotine dependence was dichotomized into low (used an e-cigarette for 1-19 days in P30D) and high (used an e-cigarette for 20–30 days).
